# Malaria risk in young male travellers but local transmission persists: a case–control study in low transmission Namibia

**DOI:** 10.1186/s12936-017-1719-x

**Published:** 2017-02-10

**Authors:** Jennifer L. Smith, Joyce Auala, Erastus Haindongo, Petrina Uusiku, Roly Gosling, Immo Kleinschmidt, Davis Mumbengegwi, Hugh J. W. Sturrock

**Affiliations:** 1 Malaria Elimination Initiative, Global Health Group, University of California, San Francisco, CA USA; 2 Multidisciplinary Research Center, University of Namibia, Windhoek, Namibia; 3 MRC Tropical Epidemiology Group, Department of Infectious Disease Epidemiology, London School of Hygiene and Tropical Medicine, Keppel Street, London, WC1E 7HT UK; 4 National Vector-Borne Disease Control Programme, Ministry of Health and Social Services, Windhoek, Namibia

## Abstract

**Background:**

A key component of malaria elimination campaigns is the identification and targeting of high risk populations. To characterize high risk populations in north central Namibia, a prospective health facility-based case–control study was conducted from December 2012–July 2014. Cases (n = 107) were all patients presenting to any of the 46 health clinics located in the study districts with a confirmed *Plasmodium* infection by multi-species rapid diagnostic test (RDT). Population controls (n = 679) for each district were RDT negative individuals residing within a household that was randomly selected from a census listing using a two-stage sampling procedure. Demographic, travel, socio-economic, behavioural, climate and vegetation data were also collected. Spatial patterns of malaria risk were analysed. Multivariate logistic regression was used to identify risk factors for malaria.

**Results:**

Malaria risk was observed to cluster along the border with Angola, and travel patterns among cases were comparatively restricted to northern Namibia and Angola. Travel to Angola was associated with excessive risk of malaria in males (OR 43.58 95% CI 2.12–896), but there was no corresponding risk associated with travel by females. This is the first study to reveal that gender can modify the effect of travel on risk of malaria. Amongst non-travellers, male gender was also associated with a higher risk of malaria compared with females (OR 1.95 95% CI 1.25–3.04). Other strong risk factors were sleeping away from the household the previous night, lower socioeconomic status, living in an area with moderate vegetation around their house, experiencing moderate rainfall in the month prior to diagnosis and living <15 km from the Angolan border.

**Conclusions:**

These findings highlight the critical need to target malaria interventions to young male travellers, who have a disproportionate risk of malaria in northern Namibia, to coordinate cross-border regional malaria prevention initiatives and to scale up coverage of prevention measures such as indoor residual spraying and long-lasting insecticide nets in high risk areas if malaria elimination is to be realized.

**Electronic supplementary material:**

The online version of this article (doi:10.1186/s12936-017-1719-x) contains supplementary material, which is available to authorized users.

## Background

Namibia has made remarkable progress along the path to malaria elimination, transitioning from a goal of reducing morbidity and mortality in 2010, to malaria elimination by 2020. This programmatic shift reflects epidemiological trends, in which reported cases declined from 562,703 in 2001 to 14,406 in 2011, and wider economic development [1]. A policy of universal coverage of insecticide treated nets (ITNs) and indoor residual spraying (IRS) in endemic areas as well as increased access to case confirmation by rapid diagnostic tests (RDTs) and treatment with artemisinin combination therapy (ACT) are likely factors contributing to this impressive decline [2].

As transmission declines, and countries such as Namibia enter the elimination phase, malaria risk becomes increasingly focused in geographic areas (hotspots) and population groups that share high risk characteristics [3–5]. To remain cost-effective, malaria control programs must undergo a shift from universal coverage of interventions, to a more tailored and targeted implementation strategy. Controlled low-endemic malaria persists in northern Namibia and, in the absence of empirical data, is anecdotally attributed to importation from Angola [1]. Critical questions remain around why some people get malaria and why malaria is confined to these border areas. Characterization of those at the highest risk of malaria may assist programmes with evidence-based targeting of interventions and improved cost-effective allocation of resources.

Geographic and demographic risk factors can be determined by analysing cross-sectional parasite rate (PR) (equivalent to infection prevalence) survey data, such as those from Malaria Indicator Surveys or Demographic and Health Surveys [6, 7]. In low transmission settings, however, the sample sizes required to power statistical analyses become operationally unfeasible, even if sensitive molecular diagnostics are used [8]. For example, the Swaziland Malaria Indicator Survey (MIS) conducted in 2010 found only one malaria case (*Plasmodium*-positive by PCR) in 4330 individuals [9]. In settings of low PR, alternative methods to cross-sectional surveys should be employed to determine risk factors associated with malaria.

One approach is to use case–control analyses to identify risk factors associated with symptomatic malaria among passively detected cases. The use of routine passive surveillance data simplifies identification of infected individuals. In lower transmission settings such as north central Namibia, the population is more likely to be immunologically naïve, making symptomatic malaria a viable proxy for transmission [10, 11]. Analysing routine case data using case–control methods provides an epidemiological tool in low malaria transmission settings where cross-sectional surveys are inappropriate. Case–control studies have been used to identify risk factors for malaria in a number of settings, including Peru [12], Columbia [13], Gambia [14] and the Kenyan Highlands [15]. More focused case–control studies have been conducted to examine associations between malaria risk and travel [16–18], risk factors for severe malaria [19, 20] as well as the relationship between malaria and bacteraemia [21].

This manuscript reports on the use of a case–control approach to examine the geographical distribution of, and key risk factors for, symptomatic malaria in Ohangwena and Omusati regions in north central Namibia, which are areas of low seasonal transmission bordering Angola. This analysis identifies characteristics that define groups at high risk of malaria. The results can inform local strategies aimed at optimizing the design and delivery of interventions to populations in this region of Namibia and perhaps more widely in southern Africa.

## Methods

### Study area

**Fig. 1:**
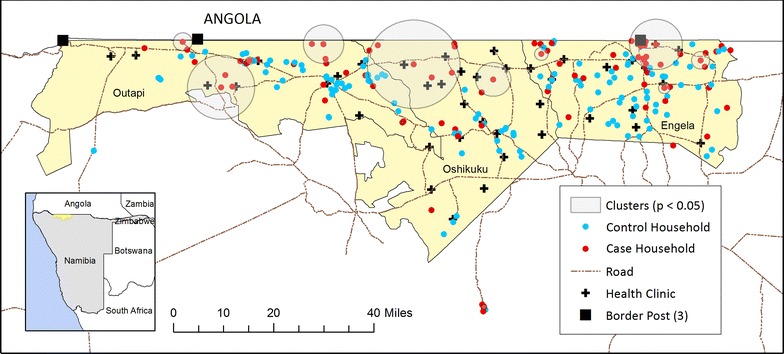
Locations of 94 passively detected cases (*red*) and 143 selected control households (*blue*) in Engela, Outapi and Oshikuku districts between December 2012–July 2014. *Circles* denote statistically significant spatial clusters of clinical malaria. *Crosses* and *squares* represent health facilities and border posts, respectively

Engela (Ohangwena region), Outapi and Oshikuku (Omusati region) are 3 of Namibia’s 34 health districts located in north central Namibia, along the border with Angola, and comprise the study area (Fig. 1). While these districts are all classified as having moderate transmission risk in Namibia’s National Strategic Plan [22], the annual parasite incidence (confirmed cases per 1000) in 2009 ranged from 4.3 in Oshikuku and 7.0 in Engela to as many as 40.4 in Outapi [23]. The region is at 780–1300 m elevation above sea level and is subject to flooding during the main rainy season (November–April), which corresponds with malaria transmission. Most roads in these predominantly rural regions are untarred dirt roads, making access difficult in some instances during the rainy season. Within the study area, there are 3 border crossings with Angola. In 2015, the study area had a reported population of approximately 375,000 [24] (16% of the total population of Namibia). The Owambo are the main ethnic group. Unemployment is common (42%) amongst adults aged over 15 years, and the main sources of household income are subsistence agriculture, pensions and, increasingly, wages and salaries [25].

The public health system in this area comprises a network of 3 hospitals, 6 health centers and 37 health facilities. The majority (74%) of health facilities nationally was reported to be government-owned in 2009 [26]. At the time of the study, the policy for first-line malaria treatment for Namibian residents who test positive for malaria by rapid diagnostic test (RDT) was artemether–lumefantrine (Coartem^®^) provided free of charge.

### Study design

A prospective case–control study, matched at the health district level, was carried out between December 2012–July 2014 in Engela, Outapi and Oshikuku health districts. Cases were individuals who were diagnosed with malaria, confirmed by multi-species RDT [CareStart™ HRP2/pLDH (Pan)], at any of the 46 government health clinics located within the study districts (Fig. 1) and resident within the study area. Population controls for each index case were consenting RDT negative individuals residing within a household randomly sampled from a census listing within the same catchment, within 2 weeks of an incident case, and not diagnosed with malaria within 1 week prior to recruitment.

A minimum sample size of 90 cases and 180 controls was required to detect an odds ratio of 2.67 (based on a 20% prevalence of exposure of interest in controls and 40% prevalence of the same exposure in cases) with 90% power and significance level of 5% (two-sided).

### Data collection

Cases and controls were visited at their households and locations recorded using GPS. All participants went through a consent procedure where the rationale and procedures of the study were explained to them. Those agreeing to participate were asked to give written consent by signature or finger print. Consent of a parent or guardian was required for those younger than age 18 years. All members of control households were screened using RDTs. A standard questionnaire was used for both cases and controls, and interviews were conducted in Oshiwambo.

Data were collected on: occupation; dichotomous measures of asset ownership; access to utilities and infrastructure (e.g. sanitation facility and source of water); housing characteristics (e.g. number of rooms for sleeping, house construction and whether there was a gap between the roof and eaves) whether the person was a guest or resident in the household; whether they slept in the household the previous night, and whether they did so under a mosquito net; information on individual or household level protection (IRS carried out in the past year, treatment-seeking for past fever, net use); travel history in the past 4 weeks (destination and duration); sleeping/spending time outside at night; presence of mosquito breeding sites in the immediate area; last time the structure was painted or re-plastered; ownership, type, age and condition of any nets (visually assessed). In addition to risk factors measured by questionnaire, digital elevation from Shuttle Radar Topography Mission, total rainfall (mm) in the month prior from the Tropical Rainfall Measuring Mission, mean enhanced vegetation index (EVI) and land surface temperature (LST) in the month prior from the Moderate Resolution Imaging Spectroradiometer instrument and distance to permanent water bodies calculated from the Global Forest Change [27] were estimated for cases and controls using Google Earth Engine [28]. Travel time to nearest facility was extracted for each household from a surface generated by Alegana et al. [29] and Euclidean distance to the Angolan border calculated in ArcMap.

Travel histories were collected for all individuals residing within case and control households, and for members from up to 4 of the closest households (neighbourhood) surveyed during reactive case detection (RACD) in Engela.

### Data processing

All responses were recorded directly into tablet computers and data uploaded on a daily basis to a centralized server. Data management and cleaning was done with R version 3.1 and statistical analysis was done with STATA version 10 (StataCorp, College Station, TX) and R. Maps were created in ArcMap version 10.1 (ESRI, Redlands, CA).

70% of travel locations were able to be assigned to a specific longitude and latitude using a number of electronic sources of information, including GeoNet Names Server [30], Google Earth [31] and Falling Grain [32]. Locations identified from one source were cross-checked against other sources and to ensure that they fell within the correct administrative area. Where possible, the remaining locations were assigned to the smallest administrative level in consultation with local officials; either constituency (10%) or region (1%). Approximately 19% of destinations could not be assigned locations, all of which were in Angola. The mean annually averaged prevalence of *Plasmodium falciparum* infection in 2–10 year olds (PfPR_2–10_) in 2013 and 2014, (estimated from the Malaria Atlas Project [33]), was extracted for each travel destination and classified into the following categories <1, 1–4.9, 5–9.9, 10–39.9 and above 40%. All destinations in Angola that were not assigned to a specific location were assigned to the 5–10% class (19%), which corresponds to areas of higher population and locations in close proximity to Namibia where geolocated travel was high. One trip within Namibia could not be assigned to a location or endemicity class and was recorded as missing.

The proportion of household members reporting travel to malaria endemic areas and to Angola was calculated for each household (excluding the individual case or control) and, in Engela, calculated for each neighbourhood (excluding those in the case/control household). This allowed distinction to be made between any added risk of infection associated with travel at the individual, household or neighbourhood level (in Engela). A scale of socioeconomic status was created using principal components analysis on household-level binary assets with frequency >5% (e.g. televisions, mobile phones, refrigerators, stoves, radio, fuel type, bicycle, vehicle, donkey and electricity) and categorical infrastructure variables (e.g. water and sanitation) [34].

Occupation was excluded from the main analysis, as this variable was only collected in the second half of the study. In addition, a high proportion of data points were missing for variables collected at the sleeping structure and net level, which included housing construction and net condition. As a consequence, these variables were not used to inform the model building process but were tested in the final model. Given the distribution of the data and to simplify analysis, binary variables were created to define (1) a “traditional home” as one with both mud walls and dirt floor, and (2) sleeping in a room with openings to the outside (any open/uncovered eaves, windows and doors) following Snyman et al. [35].

### Data analysis

Evidence for global spatial clustering (i.e. the tendency for events to occur in closer proximity to one another than expected) amongst cases was assessed by analysing the difference in K-functions between cases and controls using the spatstat package in R [36], and simulating confidence envelopes under the assumption of complete spatial randomness. Local clusters of malaria were identified using Kulldorff’s scan statistic in SaTScan version 9.4.2. The analysis was run separately for each of the 3 health districts, as controls were recruited within the same district as incident cases arose.

Potential risk factors were assessed by logistic regression using generalized estimating equations (GEE) in STATA. All models included a dummy indicator for district (matching variable) and accounted for correlation between observations within households using an exchangeable correlation structure and robust variance estimator. An adjusted logistic regression model was built by adding factors one at a time, based on the unadjusted associations, and retaining those covariates which improved the quasi-likelihood under the independence model criterion (QIC) or materially confounded the effect of other factors included in the model (changed the odds ratio estimate by more than 15%). Controls included all RDT-negative individuals identified within control households, to increase the power of the study. Non-linear relationships with continuous covariates were assessed by including a squared term in the regression model or categorizing covariates according to breaks, based on a fitted ‘lowess’ curve. Based on observed differences in the age-gender profile for cases between Engela and Omusati/Oshikuku and the potentially differing epidemiology of malaria in these settings, interactions were tested in the final model between (1) study area and age and gender, (2) reported travel and gender and (3) reported travel and coverage of long-lasting insecticide nets (LLIN) and IRS. Interactions retained in the final model were calculated using post-estimation in STATA and presented following the STROBE (STrengthening the Reporting of OBservational studies in Epidemiology) recommendations [37]. Interaction effects on the multiplicative scale were estimated from the regression model and calculated from log-odds on the additive scale using ‘nlcom’ in STATA. Spatial dependency was checked by visually inspecting semi-variograms of the residuals from the full multivariate model, averaged at the household level.

Two sensitivity analyses were carried out (Additional file 1: Table S1). First, as it has been shown that individuals who live further from health facilities are less likely to seek treatment [29], the potential for selection bias introduced by using passively detected cases and population controls was evaluated. Controls were re-sampled according to their predicted probability of seeking treatment for fever [29] for the first sensitivity analysis, ensuring that controls were representative of the same population from which cases arose. A second sensitivity analysis evaluated the impact of differences in the temporal enrollment of cases and controls, and resampled controls to generate a subset of RDT-negative individuals from households matched by transmission season (high *vs* low).

### Ethical approval

Institutional Review Board approval for this study was obtained from the University of California San Francisco, the University of Namibia and the Ministry of Health and Social Services of Namibia.

## Results

### Descriptive analysis

Between December 2012 and June 2014, 133 RDT-confirmed malaria cases were successfully traced to their households and enrolled in the study. It was not known what fraction of cases that reported to facilities were successfully followed up in Outapi and Oshikuku, although it is likely to be similar to Engela, where 70/138 (50.7%) of eligible individuals were successfully followed up. Simultaneously, 889 community controls from 147 randomly selected control households were surveyed between February 2013 and July 2014. The proportion not present at follow-up was similar among cases (18.8%) and controls (16.6%) (Additional file 2: Table S2). These individuals were more likely to be male (57.9%) and older (median age 18 IQR 13.0–42.0 years) than non-missing individuals (44.8%, median age 16 IQR 7.0–21.0 years) (p < 0.0001). Consent refusal rates among those present were low for both cases and controls (0.9 and 6.8%, respectively, Additional file 2: Table S2) although they did statistically differ (p = 0.023). Eleven individuals missing an RDT outcome and four controls with a positive RDT test (all males from Outapi and ranging from 2 to 50 years of age) were excluded from further analysis.

**Fig. 2:**
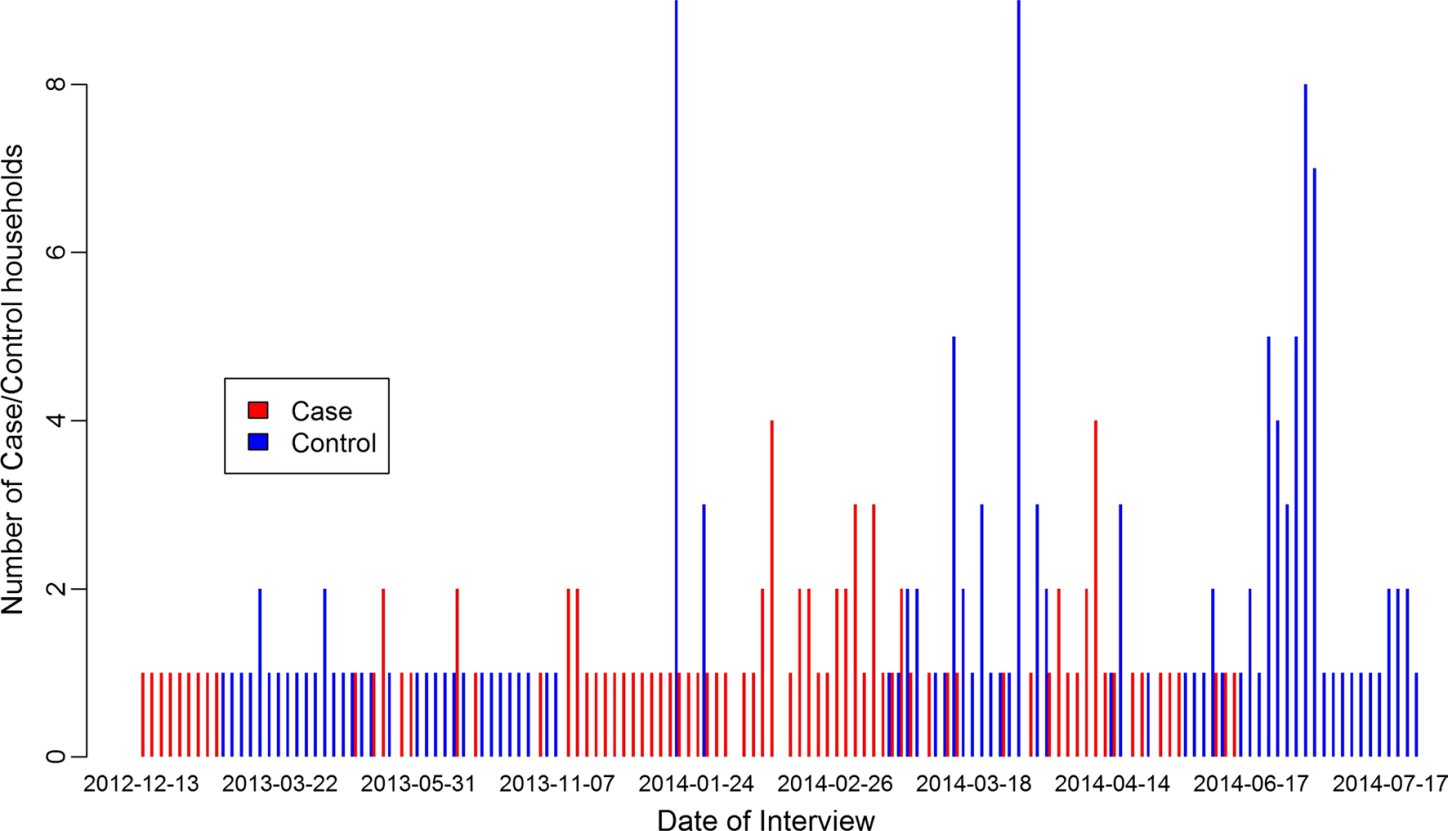
Temporal recruitment of cases and controls between December 2012 and July 2014

The enrollment of cases and controls over time is shown in Fig. 2. Table 1 shows the demographics of study participants included in the analysis (107 cases and 679 controls). The case to control household ratio was 1:1.3 and inclusion of all control household members provided an average of 6.3 controls per case. The clustered exposures of controls within a household reduced the effective sample size by fourfold based on the design effect for overnight travel.

**Table 1:** Demographic and intervention characteristics of 786 study participants recruited in Engela (December 2012–July 2014) and Omusati and Oshikuku (January 2014–July 2014)

Variable	Engela		Omusati/Oshikuku	
	Cases n = 57 (%)	Controls n = 298 (%)	Cases n = 50 (%)	Controls n = 381 (%)
Sex				
Female	18 (31.6)	174 (58.4)	26 (52.0)	216 (56.7)
Male	39 (68.4)	124 (41.6)	24 (48.0)	165 (43.3)
Age (years)				
0–4	6 (10.5)	37 (12.4)	4 (8.0)	67 (17.6)
5–14	13 (22.8)	91 (30.5)	12 (24.0)	127 (33.3)
15–24	26 (45.6)	81 (27.2)	14 (28.0)	73 (19.2)
25–34	8 (14.0)	36 (12.1)	7 (14.0)	38 (10.0)
35–44	2 (3.5)	20 (6.7)	7 (14.0)	19 (5.0)
45+	2 (3.5)	31 (10.4)	6 (12.0)	34 (8.9)
Missing	0 (0.0)	2 (0.7)	0 (0.0)	23 (6.0)
House sprayed in past year	14 (24.6)	109 (36.6)	14 (28.0)	68 (17.8)
Missing	1 (1.8)	1 (0.3)	0 (0.0)	2 (0.5)
Slept under net previous night	16 (28.1)	42 (14.1)	21 (42.0)	106 (27.8)
Missing	0 (0.0)	4 (1.3)	0 (0.0)	5 (1.3)
Travel to endemic areas (≥1 PfPR2-10)^a^	21 (36.8)	25 (8.4)	5 (10.0)	33 (8.7)

PfPR2-10: mean annually averaged prevalence of P falciparum infection in 2–10 year olds

^a^Average of 2013 and 2014

The demographic profile and travel history of cases and controls varied between Engela and the other two districts, Outapi and Oshikuku, and likely confounded unadjusted estimates of association (Table 1). Reported spray coverage and net use were generally very low (mean 26.2 and 23.8%), with no clear pattern between cases and controls (Table 1).

### Spatial clustering of malaria and travel patterns

The distribution of malaria cases was clustered in the study area (K-function p = 0.02) and 9 “hot-spots” ranging in size from 1 to 30 households were detected, after accounting for the spatial distribution of population controls (Fig. 1). Clusters were predominantly located along the border with Angola.

**Fig. 3:**
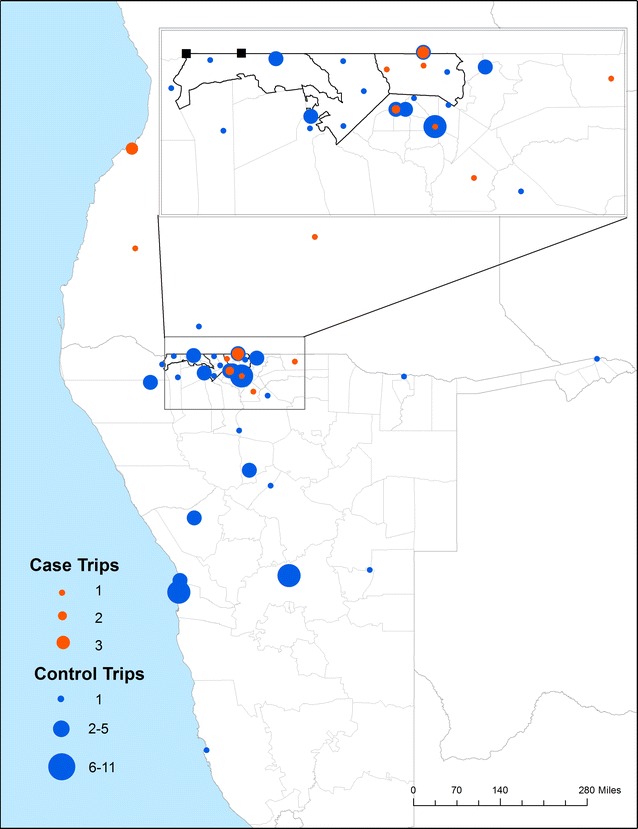
Travel destinations of 84 geolocated trips, where the *color* and *size of the dots* denote the number of cases (*orange*) or controls (*blue*) that travelled to that location

In total, 113 trips were reported by 111 (14.1%) individuals included in the study (Table 2). Almost all (98.3%) individuals took only 1 trip, and travel by cases was more frequently of short (1–13 days) or long (1–6 months) duration compared with travel by controls. Within the study region, cases and controls had similar travel patterns which included relatively frequent travel to local commercial centres such as Oshakati, Ondangwa and Onambango (Table 2; Fig. 3). Outside of the study area, controls commonly travelled to southern Namibia, including urban centers such as Windhoek and Walvis Bay. Cross-border travel to Angola made up the majority (81.0%) of travel by cases in Engela, but represented a much smaller proportion of travel by controls (12.2%). Cases and controls in Omusati and Oshikuku had more similar travel patterns to locations in Namibia (57.1 and 71.4%, respectively) and Angola (42.9 vs 28.6%). A larger proportion of trips were taken by males among cases (71.4%) than controls (41.0%).

**Table 2:** Travel characteristics of cases and controls reporting any overnight travel in the 6 weeks prior to diagnosis

Travel characteristic	Engela		Omusati/Oshikuku	
	Cases n = 57 (%)	Controls n = 298 (%)	Cases n = 50 (%)	Controls n = 381 (%)^a^
Total number of travelers	21 (36.8)	41 (13.8)	7 (14.0)	42 (11.0)
Number with more than 1 trip	0 (0.0)	2^b^ (0.7)	0 (0.0)	0 (0.0)
Male traveler	16 (28.1)	19 (6.4)	4 (8.0)	15 (3.9)
Age of traveler (years)				
<5	1 (1.8)	5 (1.7)	1 (2.0)	10 (2.6)
5–14	4 (7.0)	7 (2.3)	0 (0.0)	1 (0.3)
15–29	12 (21.0)	15 (5.0)	3 (6.0)	16 (4.2)
30–44	3 (5.3)	10 (3.4)	0 (0.0)	11 (2.9)
45–59	1 (1.8)	2 (0.7)	3 (6.0)	0 (0.0)
60+	0 (0.0)	2 (0.7)	0 (0.0)	3 (0.8)
Endemicity class (PfPR2-10)				
Namibia (%)	4 (7.0)	36 (12.1)	4 (8.0)	30 (7.9)
<1	0 (0.0)	16 (5.4)	2 (4.0)	9 (2.4)
1–4.9	1 (1.8)	2 (0.7)	1 (2.0)	4 (1.0)
5–10	3 (5.3)	18 (6.0)	1 (2.0)	17 (4.5)
Angola (%)	17 (29.8)	5 (1.7)	3 (6.0)	12 (3.1)
<1	0 (0.0)	0 (0.0)	0 (0.0)	0 (0.0)
1–4.9	6 (10.5)	0 (0.0)	0 (0.0)	1 (0.3)
5–10	11^c^ (19.3)	5^d^ (1.7)	3^e^ (6.0)	11^f^ (2.9)
Travel to higher endemic class	0 (0.0)	16 (5.4)	4 (8.0)	18 (4.7)
Duration of stay				
1–13 days	6 (10.5)	7 (2.3)	1 (2.0)	3 (0.8)
2–4 weeks	3 (5.3)	11 (3.7)	1 (2.0)	6 (1.6)
1–6 months	12 (21.0)	23 (7.7)	5 (10.0)	33 (8.7)

PfPR2-10: mean annually averaged prevalence of P falciparum infection in 2–10 year olds

^a^One control missing age

^b^One female and one male took two and three trips respectively. All trips were of the same duration (1–13 days) and to destinations in Namibia. Travel was classified based on the highest endemicity destination: PfPR2-10 < 1% (four trips) or PfPR2-10 1–4.9% (one trip)

Number of missing trip coordinates in Angola and assumed to lie in PfPR2-10 5–10%: 6^c^; 3^d^; 3^e^; 9^f^

**Fig. 4:**
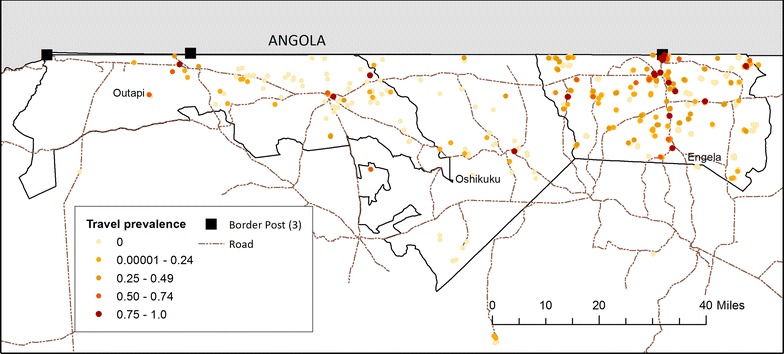
Prevalence of reported overnight travel within the last 6 weeks in 94 case and 143 control households

Visually, travel prevalence at the household level had a more focal distribution in Outapi and Oshikuku compared with Engela, where travel was more common and covered longer distances. Households with higher prevalence of travel appeared to cluster in proximity to border posts and main roads, and in particular around Omahanene Border Post in Engela (Fig. 4).

### Risk factors for malaria

The unadjusted odds of malaria for all potential risk factors analysed are shown in Additional file 3: Table S3, and the adjusted odds for those retained in the full model are presented in Tables 3 and 4. Included covariates explained all spatial autocorrelation, suggesting that important spatial risk factors were adjusted for.

**Table 3:** Modification of the effect of travel to Angola on malaria by gender in unadjusted and adjusted analyses

	No travel to Angola		Travel to Angola		ORs (95% CI) for travel within gender strata
	N with/without malaria	OR (95% CI)	N with/without malaria	OR (95% CI)	
Unadjusted OR^a^					
Female	34/352	1.0	3/13	1.14 (0.88–1.47)	1.14 (0.88–1.47)
Male	46/266	1.10 (1.04–1.17)	15/3	4.97 (1.59–15.52)	4.50 (1.45–14.01)
Adjusted OR^b^					
Female	34/352	1.0	3/13	1.65 (0.36–7.47)	1.65 (0.36–7.47)
Male	46/266	1.95 (1.25–3.04)	15/3	85.00 (4.16–1736)	43.58 (2.12–896)

Measure of effect modification on additive scale: RERI (95% CI) 82.39 (−174 to 339); P = 0.53. Confidence intervals calculated using Delta approximation

Measure of effect modification on multiplicative scale: ratio of ORs (95% CI) 26.46 (0.92–766); P = 0.05

^a^GEE adjusted for health district (matching variable) and clustering of controls within households

^b^ORs are adjusted for all covariates listed in Table 4

**Table 4:** Unadjusted and adjusted odds ratios from bivariate and multivariate logistic regression

Variable	Number^a^		Unadjusted OR (95% CI)	P value	Adjusted OR (95% CI)	P value
	Case N = 98	Control N = 634				
Age group (years)						
<5	9	100	1	–	1	–
5–14	23	210	1.13 (1.00–1.28)	0.06	1.48 (0.65–3.36)	0.35
15–29	37	150	1.22 (1.07–1.38)	0.002	2.14 (0.99–4.63)	0.05
30–44	14	73	1.05 (0.89–1.26)	0.52	2.23 (0.87–5.72)	0.10
45–59	8	38	1.05 (0.80–1.37)	0.74	3.03 (1.11–8.27)	0.03
60+	7	63	0.90 (0.77–1.05)	0.18	1.24 (0.55–2.80)	0.61
Location slept previous night						
In household	87	624	1	–	1	–
Away from household	11	10	2.46 (1.35–4.48)	0.003	5.61 (1.97–16.02)	0.001
Higher socioeconomic status^b^	98	634	0.78 (0.62–0.98)	0.03	0.65 (0.47–0.91)	0.01
Squared term	–	–	1.10 (1.00–1.19)	0.03	1.13 (1.01–1.27)	0.04
Predicted travel time to clinic						
0–4	52	392	1	–	1	–
5–14	43	120	2.51 (1.34–4.70)	0.004	2.07 (0.86–5.01)	0.11
15–46	3	122	0.58 (0.15–2.33)	0.44	0.05 (0.005–0.59)	0.02
More than 15 km from Angolan border	23	298	1	–	1	–
<15 km from Angolan border	75	336	4.34 (1.91–9.85)	<0.0001	2.86 (1.17–6.97)	0.02
Enhanced Vegetation Index (EVI)^b^						
0.11–0.24	37	443	1	–	1	–
0.25–0.34	54	145	5.32 (2.49–11.38)	<0.0001	14.62 (3.73–57.27)	<0.0001
0.35–0.45	7	46	1.70 (0.43–6.71)	<0.0001	2.00 (0.23–17.20)	0.53
Total rainfall in prior month (mm)						
0–19	14	207	1	–	1	–
20–39	63	171	7.25 (3.33–15.79)	<0.0001	2.79 (1.01–7.70)	0.05
40–67	21	256	2.14 (0.87–5.31)	0.10	0.25 (0.06–0.99)	0.05
District: Engela	51	277	1	–	1	–
Oshikuku^c^	21	74	1.54 (0.62–3.81)	0.94	5.06 (1.35–18.97)	0.02
Outapi	26	283	0.50 (0.21–1.16)	0.11	1.13 (0.45–2.87)	0.79

All GEE adjusted for health district (matching variable) and clustering of controls within households. Multivariate GRR adjusted for the interaction effect of gender and travel in Table 3

*OR* odds ratio, *GEE* generalized estimating equations, *CI* confidence interval, *QIC* quasilikelihood under the independence model criterion, *m* meters, *°C* degrees Celsius, *mm* millimeters, *km* kilometers

^a^Numbers restricted to non-missing data in final multivariate model; full bivariate analyses included in Table S3

^b^Socioeconomic measure is first component of the PCA, included as a continuous measure

^c^The higher adjusted odds of malaria observed in Oshikuku is attributed to a lower number of controls recruited per case in this area compared to the other districts

### Demographic risk factors

The adjusted odds of malaria in participants aged 15–29 years (OR 2.14 95% CI 0.99–4.63) and 45–59 years (OR 3.03 95% CI 1.11–8.27) were higher than those age <5 years. When the analyses were restricted to controls who were resampled based on the probability of reporting to a health facility for fever, the age groups 5–14 years and 15–30 years were at highest risk of malaria compared with children <5 years (Additional file 4: Table S4; Additional file 5: Table S5).

This may reflect differing reporting rates between age groups by distance to the health facility (i.e. families who live near to a health facility may be more likely to attend with children <5 years of age).

### Travel-related risk factors

The effect of travel on the odds of being a case was modified by gender (Table 3). The adjusted odds of malaria in males was twice as high as females amongst non-travellers (OR 1.95 95% CI 1.25–3.04), and male travellers had an added gender-specific risk associated with travel: male travellers to Angola had a large increased risk of malaria compared with male non-travellers (adjusted OR 43.58 95% CI 2.12–896) and female travellers to Angola (adjusted OR 85.00 95% CI 4.16–1736). Index cases had no added risk of malaria associated with household or neighborhood-level prevalence of travel.

### Location-related risk factors

The adjusted odds of malaria were more than twice as high for individuals living <15 km from the Angolan border (adjusted OR 2.86 95% CI 1.17–6.97). Moderate levels of vegetation (EVI between 0.25 and 0.34) in the prior month were associated with a higher adjusted odds of malaria compared with areas of more sparse vegetation (OR 14.62 95% CI 3.73–57.27) and moderate rainfall (20–39 mm) in the preceding month was associated with a higher risk of malaria, compared with zero-to-low rainfall (adjusted OR 2.79 95% CI 1.01–7.70). By contrast, heavier rainfall (40 mm and more) was protective (adjusted OR 0.25 95% CI 0.06–0.99). However, the association of total rainfall in the preceding month shifted to borderline significance in the sensitivity analysis that re-sampled controls matched on transmission season (Methods-Data Analysis). The higher adjusted odds of malaria observed in Oshikuku is attributed to a lower proportion of controls recruited from this district (and lower control to case ratio), compared to the other districts.

### Bed net ownership and IRS coverage

No associations between IRS or bed net coverage and malaria were observed in the adjusted or unadjusted analyses. While a high proportion of case and control households had at least one net per household (73.8 vs 65.2%), notably fewer households met the target coverage of one net for every two people (31.8 vs 23.4%) (Additional file 3: Table S3).

### Socioeconomic factors and access to health services

Higher socioeconomic status was strongly protective against malaria (adjusted OR 0.65 95% CI 0.47–0.91) using the first principal component, which weighted ownership of electronic assets and infrastructure variables relatively highly. Residing more than 15 min travel time from a health clinic was also associated with a lower risk of malaria compared with those living within 5 min travel time (adjusted OR 0.05 95% CI 0.005–0.59). However, this relationship dropped out during the sensitivity analyses when controls were resampled according to their predicted probability of seeking treatment for fever. This suggests that there may be selection bias present in the original control sample (i.e. the population-based sample of controls may not be representative of the population from which the cases arose).

An analysis of the subset of data that had information on housing design showed a higher risk of malaria associated with ever having painted or re-plastered the walls compared to never painting/plastering (adjusted OR 3.02 95% CI 1.87–4.88), after adjusting for factors included in the full model. A borderline protective effect of window covers or glass windows in the sleeping structure compared with no windows was also observed (adjusted OR 0.67 95% CI 0.44–1.02, p = 0.07). No effect of type of home construction nor sleeping in structures open to the outside was seen, possibly due to very low sample size of traditional houses or those closed to the outside.

Occupational data were available for a subset of 23 cases and 120 controls surveyed in Engela after February 2014. The small sample size precluded running the full adjusted analyses, but there was evidence that individuals employed in small market sales and trade were more likely to be a case (OR 37.26 95% CI 2.72–510) after adjusting for age, gender and distance from the Angolan border.

### Behavioural risk factors

Sleeping away from the household the previous night was associated with higher risk of malaria (adjusted OR 5.61 95% CI 1.97–16.02). Sleeping under a net the previous night and treatment for previous fever (a marker for diagnosis as a case at the health facility) were not associated with malaria in the adjusted analysis.

## Discussion

This case–control study clearly shows that young males between the ages of 15–29 years travelling to Angola are at excessive risk of getting malaria and presenting to health facilities in northern Namibia. This is important because importation of malaria that results in re-introduction of malaria is probably the biggest barrier to countries successfully eliminating malaria and staying malaria free [38–42]. Characterizing the population at risk of importing malaria, as has been done in this study, will help the malaria control programme target interventions appropriately. In addition, this study clearly identified that malaria risk is spatially clustered suggesting geographical hotspots of malaria transmission and ongoing local transmission related to environmental receptivity. The malaria control programme of Namibia now has evidence based information to support policy formulation for managing malaria importation by high-risk populations and cross-border collaboration with Angola to reduce its malaria burden, as well as re-focusing energies to wipe out remaining foci of transmission and prevent re-introduction.

Travel [9, 17, 18] and being male [16, 17, 43] have previously shown to be associated with increased risk of malaria in low endemic and elimination settings [4]. This study provides the first evidence that gender can modify the effect of travel on malaria risk. The tendency for travelling men to be at increased risk may be due to travel to particular areas or other gender-specific behaviour (e.g. less use of preventive measures, more time spent outdoors at night) while travelling that increase exposure to mosquitoes. Short-term cross border movements related to visiting family, school attendance and trade are common in the study area because the local ethnic group, the Owambo, live on both sides of the Namibia/Angola border. However, the findings from this study suggest that high risk travel to Angola is by men in particular and likely in relation to trade.

Living within 15 km of Angola was an independent risk factor for malaria after controlling for individual travel and environmental covariates, and clusters of cases were located along the border. These findings suggest the presence of an unknown risk factor associated with malaria receptivity or some diffusion of risk from Angola via mosquitoes or human movement. Household and community-level travel was not found to be a risk factor in this study, suggesting that vector movement may be more important. Coordinating cross-border vector control campaigns and focusing efforts on achieving high IRS coverage within this area may be a useful strategy in this context.

This study also identified some well-known risk factors for malaria transmission that present on-going challenges for the malaria programme to tackle. Climatic factors including moderate vegetation (EVI) (a measure of relative humidity [44]) and moderate (20–39 mm) rainfall in the month prior to diagnosis suggest that receptivity persists in distinct geographies, further evidenced by the geographical clustering of cases found in the analysis [23, 45]. Too much rain (>39 mm) probably leads to breeding sites being flushed of larvae and too little rain probably results in few breeding sites for the vectors [46, 47]. After accounting for rainfall, higher levels of EVI may relate to greater availability of larval habitats [48]. Other factors identified associated with increased malaria risk include lower socioeconomic status, sleeping away from the household the previous night, and borderline higher risk in individuals aged between 15 and 29 years. IRS coverage and LLIN use were low (mean 25.1 and 24.2%) in both cases and controls. Ensuring that the geographical “hotspots” and people of lower socio-economic status are protected by vector control may go some way in reducing receptivity and preventing local malaria transmission.

The findings from this study have programmatic implications. A full quarter (25.8%) of cases passively detected in Engela resided in Angola and were ineligible for the study. The importance of travel to and living near Angola emphasizes the need for stronger cross-border collaboration. Regional and cross-border initiatives, such as the recently launched Elimination 8 in southern Africa [49] and Trans Kunene Malaria Initiative [50] attempt to tackle cross-border importation and target regional sources of infections. Male travellers, identified in this study as importers of malaria, may be less likely to seek health care and could benefit from targeted services to improve case detection and malaria prevention. Providing testing, treatment and prevention at specific local commercial centers or border posts may be an effective approach to prevent young men from spreading malaria to local communities. Low levels of LLIN use and IRS coverage indicate a need for stronger distribution systems, health education and improved targeting to high risk populations and high risk geographies. Households with higher prevalence of travel appeared to cluster in proximity to border posts and main roads, and in particular around the Omahanene Border Post in Engela. In receptive areas, such “travel hotspots” would be priority areas to improve interventions coverage, including better vector control, and could be defined using population-based travel surveys. This study also found that covered or glass windows, a relatively minor housing improvement, may have a beneficial impact on transmission. This is consistent with an increasing body of evidence that supports that use of modern construction materials and closed housing designs are protective against clinical malaria and mosquito density [14, 35, 51–53].

A number of limitations due to study design and implementation are noted. First, cases are passively detected and may have a higher likelihood of reporting to a health facility than population-based controls. The results from the sensitivity analysis, which resampled controls according to probability of seeking treatment, did suggest that selection bias was present but that most associations in the model were unchanged. Second, the study did not capture (1) cases who were not resident in the study districts, (2) a high proportion of eligible cases who were not able to be traced (50.7%) and (3) individuals who were absent from their homes during the time of survey. While eligible cases that were lost to follow up had a similar demographic profile as included cases, the gender distribution of passively detected cases resident in Angola had a higher proportion of females (45.5 and 54.6%, p = 0.03). Absent individuals were more likely to be males and older than non-missing individuals, fitting the high risk profile in this setting. As a result, these analyses may have underestimated the importance of cross-border importation and domestic travel by males in this context. Third, passive detection of cases may miss certain high risk populations who do not report to health facilities. For example, anecdotal evidence suggests that some Angolan children attend boarding schools in Namibia during the week and return home on the weekend, and may be less likely to report to Namibian health facilities. Fourth, the study design contains a number of sources of potential measurement error, including recall bias for self-reported measures of net usage and travel history, as well as uncertainty around the relevant period of exposure due to variation in the length of time between infection and diagnosis. A 1-month temporal lag for environmental data may be too short to capture all ecologically important conditions, however aggregate measures can mask intensity of individual rainfall events, which could have an important impact on mosquito larvae by flushing them out of their habitats [54]. Further misclassification may be introduced by geolocation errors, particularly of travel locations in Angola, and use of modelled endemicity maps. Fifth, the low diagnostic sensitivity of RDTs could dilute any differences between cases and controls but given the very low transmission in this setting, it is likely that only a very small number of infections among the control group would have been negative by RDT. Finally, the statistical power was limited by the relatively small samples size, leading to wide confidence intervals around low prevalence exposures, such as travel.

The use of a case–control methodology allowed the study team and the malaria control programme to gain useful information that will guide programmatic implementation of interventions that would not have been possible using a malaria indicator survey. The methods used in this study could be improved and simplified by recruiting cases and controls in the health facilities. This modification would: allow us to capture cases who reside outside of the study area or who are difficult to trace, reduce the effects of absenteeism in both cases and control arms; reduce bias in differences between case selected at health facilities and community controls; and eliminate the delay in seeking controls in the community due to high malaria case burden during the malaria season. Limitations will persist, for example: in very low transmission settings recruitment is likely to occur over a large area leading to the loss of granularity of risk factors and recruitment may continue over several transmission seasons missing subtle temporal risks. Malaria programmes can complement case–control studies with other more targeted survey approaches that focus on high risk populations such time-location and social network sampling [55], in order to gain more comprehensive data on who, where and how to target interventions in the last mile of malaria elimination.

## Conclusions

In this low endemic, cross-border setting, travel to Angola was found to be the strongest risk factor for malaria in males, but not females. Furthermore, cases had distinct travel patterns when compared with the general population. Even on this relatively small scale, moisture variables (e.g. rainfall and EVI) were important climatic drivers of malaria transmission. Together, these findings suggest the need to address importation by male travellers as well as prevent local transmission within highly receptive foci in the study area. Specifically, these findings can be used to target border screening strategies and increase intervention coverage, including vector control, amongst this high-risk group and in geographical hotspots of infection and travel. Future work is focused on developing a standardized set of surveillance tools building on this approach.

## Additional files

**Additional file 1.** Power calculations for case–control analyses using the full dataset and restricted, re-sampled controls.

**Additional file 2.** Exclusions from the dataset due to lack of consent or missing RDT test results in control households.

**Additional file 3.** Univariate analysis of main risk factors for malaria using GEE adjusted for health district (matching variable) and clustering of controls within households.

**Additional file 4.** Sensitivity analysis for modification of the effect of travel to Angola on malaria by gender in adjusted analyses^1^.

**Additional file 5.** Sensitivity analysis for multivariate logistic regression model^1^.
