# Supplementary material for: Malaria risk in young male travellers but local transmission persists: a case–control study in low transmission Namibia
Source: Malar J. 2017 Feb 10;16:70. doi: 10.1186/s12936-017-1719-x (PMC5303241; doi:10.1186/s12936-017-1719-x)
Supplement: Supplementary file 1 — Additional file 1. Power calculations for case–control analyses using the full dataset and restricted, re-sampled controls. [file 12936_2017_1719_MOESM1_ESM.docx]

| Table S1. Power calculations for case-control analyses using the full dataset and restricted, re-sampled controls. | | | | |
| --- | --- | --- | --- | --- |
| **Control Group** | **Description** | **Sample size** | **Power^1^** | **Potential Biases** |
| Full dataset | All RDT negative individuals within randomly selected control households | Cases: 107  Controls: 679 Control HH: 143 | 97% | Differences in exposure distribution due to time; differences in probability of reporting to health facility; differences between period of exposure assessment |
| Sensitivity Analysis 1 | A subset of the full dataset, including RDT negative individuals from 119 control households resampled according to the probability of presenting to a health facility. | Cases: 107  Controls: 488  Control HH: 119 | 96% | Differences in exposure distribution due to time; differences between period of exposure assessment; unmeasured individual-level differences in probability of reporting |
| Sensitivity Analysis 2 | A subset of the full dataset, including RDT negative individuals from control households, frequency matched by transmission season | Cases: 107  Controls: 635  Control HH: 121 | 97% | Differences in probability of reporting to health facility; differences between period of exposure assessment |
| ^1^ Power to detect a difference in proportions corresponding to an odds ratio of 2.67 (p1=0.2 and p2=0.4); calculations adjusted for observed design effect of 2 for history of travel. | | | | |
