# Supplementary material for: Malaria risk in young male travellers but local transmission persists: a case–control study in low transmission Namibia
Source: Malar J. 2017 Feb 10;16:70. doi: 10.1186/s12936-017-1719-x (PMC5303241; doi:10.1186/s12936-017-1719-x)
Supplement: Supplementary file 2 — Additional file 2. Exclusions from the dataset due to lack of consent or missing RDT test results in control households. [file 12936_2017_1719_MOESM2_ESM.docx]

| Table S2. Exclusions from the dataset due to lack of consent or missing RDT test results in control households. | | | | | | | |
| --- | --- | --- | --- | --- | --- | --- | --- |
|  | **Cases**  **Number (%)** |  | **Controls**  **Number (%)** |  | **Total**  **Number (%)** |  | **P value^1^** |
| Recruitment & Consent | | | | | | | |
| Total enumerated | 133 (100)^2^ |  | 889 (100)^3^ |  | 1022 (100) |  | - |
| Not present | 25 (18.8) |  | 148 (16.6) |  | 173 (16.9) |  | 0.54 |
| Total present | 108 (100) |  | 741 (100) |  | 849 (100) |  | - |
| Refused consent | 1 (0.9) |  | 47 (6.3) |  | 48 (5.6) |  | 0.02 |
| RDT results at diagnosis | | | | | | | |
| Total consented | 107 (100) |  | 694 (100) |  | 801 (100) |  | - |
| RDT not done | 0 (0.0) |  | 10 (1.4) |  | 10 (1.2) |  | 0.37 |
| RDT not valid | 0 (0.0) |  | 1 (0.1) |  | 1 (0.0) |  | 1 |
| Discordant RDT results^4^ | 0 (0.0) |  | 4 (0.6) |  | 4 (13.9) |  | 1 |
| RDT: rapid diagnostic test; RACD: reactive case detection  ^1^ P-value from Fisher's Exact Test  ^2^ Index cases from 120 households  ^3^ All individuals from 147 index control households  ^4^ RDT status at the time of diagnosis | | | | | | | |
