# Supplementary material for: Malaria risk in young male travellers but local transmission persists: a case–control study in low transmission Namibia
Source: Malar J. 2017 Feb 10;16:70. doi: 10.1186/s12936-017-1719-x (PMC5303241; doi:10.1186/s12936-017-1719-x)
Supplement: Supplementary file 3 — Additional file 3. Univariate analysis of main risk factors for malaria using GEE adjusted for health district (matching variable) and clustering of controls within households. [file 12936_2017_1719_MOESM3_ESM.docx]

| Table S3. Univariate analysis of main risk factors for malaria using GEE adjusted for health district (matching variable) and clustering of controls within households^1^ | | | | | | | | | |
| --- | --- | --- | --- | --- | --- | --- | --- | --- | --- |
|  | | | ^Number of cases (%)^  ^N=107^ | ^Number of controls (%)^  ^N=679^ | | | Crude odds  ratio (95% CI) | | P value^2^ |
| Individual level | | | | | | | | | |
| Male | | | 63 (*58.9*) | | 289 (*42.6*) | | 1.21 (1.10-1.33) | | <0.0001** |
| Age group (years, 25 missing)^2^ | | |  | |  | |  | |  |
|  | | <5 | 10 (9.4) | | 104 (15.9) | | 1 | |  |
|  | | 5-14 | 25 (23.4) | | 218 (33.3) | | 1.11 (0.99-1.24) | | 0.08* |
|  | | 15-29 | 40 (37.4) | | 154 (23.6) | | 1.20 (1.07-1.35) | | 0.003** |
|  | | 30 -44 | 15 (14.0) | | 74 (11.3) | | 1.05 (0.89-1.23) | | 0.55 |
|  | | 45-59 | 9 (8.4) | | 39 (6.0) | | 1.05 (0.81-1.35) | | 0.71 |
|  | | 60+ | 8 (7.5) | | 65 (9.9) | | 0.91 (0.79-1.06) | | 0.24 |
| Guest in household (27 missing) | | | 1 (1.0) | | 12 (1.8) | | 0.80 (0.54-1.16) | | 0.24 |
| Slept under net previous night (9 missing) | | | 37 (34.6) | | 148 (22.1) | | 1.16 (0.94-1.43) | | 0.16 |
| Did not sleep in household previous night  (2 missing) | | | 12 (11.2) | | 10 (1.5) | | 2.51 (1.37-4.58) | | 0.003** |
| Outside the previous night (1 missing) | | | 32 (29.9) | | 107 (15.8) | | 1.22 (1.03 -1.43) | | 0.02** |
| Outside at night: sleeping (2 missing) | | | 4 (3.7) | | 4 (0.6) | | 2.13 (0.82-5.56) | | 0.12 |
| Outside at night: other (10 missing) | | | 32 (29.9) | | 106 (15.8) | | 1.21 (1.02-1.43) | | 0.03** |
| Travel: No travel / Travel <1% PfPR2-10 | | | 81 (75.7) | | 621 (91.5) | | 1 | |  |
|  | Travel to any endemic area (≥1% PfPR2-10) | | 26 (24.3) | | 58 (8.5) | | 1.65 (1.25-2.17) | | <0.0001** |
| Travel: No travel / Travel <1% PfPR2-10 | | | 81 (75.7) | | 621 (91.5) | | 1 | |  |
|  | Travel to low endemic (1-5% PfPR2-10) | | 8 (7.5) | | 7 (1.0) | | 2.76 (1.42-5.33) | | 0.003* |
|  | Travel to mod. endemic (5-10% PfPR2-10) | | 18 (16.8) | | 51 (7.5) | | 1.45 (1.09-1.92) | | 0.01* |
| Travel to Angola | | | 20 (18.7) | | 17 (2.5) | | 3.00 (1.74-5.14) | | <0.0001** |
| Duration of travel | | |  | |  | |  | |  |
|  | | No travel | 79 (73.8) | | 596 (87.8) | | 1 | |  |
|  | | 1 – 14 days | 7 (6.5) | | 10 (1.5) | | 1.81 (1.06-3.09) | | 0.03** |
|  | | 15 – 30 days | 4 (3.7) | | 17 (2.5) | | 1.13 (0.79-1.61) | | 0.52 |
|  | | 1 – 6 months | 17 (15.9) | | 56 (8.2) | | 1.37 (1.04-1.79) | | 0.02** |
| Night outside district in last six weeks  (2 missing) | | | 31 (29.0) | | 84 (12.4) | | 1.48 (1.19-1.85) | | 0.001** |
| Treated for last fever (1 missing)^3^ | | |  | |  | |  | |  |
|  | | No fever | 14 (13.1) | | 384 (56.6) | | 1 | |  |
|  | | Fever not treated | 0 (0.0) | | 105 (15.5) | | - | | - |
|  | | Fever treated | 93 (86.9) | | 189 (27.9) | | 4.15 (3.04-5.68) | | <0.0001** |
| Sleeping structure level | | | | | | | | | |
| Window type (202 missing) | | |  | |  | | |  |  |
|  | | No windows | 47 (53.4) | | 174 (35.1) | | | 1 | - |
|  | | No cover | 9 (10.2) | | 47 (9.5) | | | 0.89 (0.72-1.10) | 0.28 |
|  | | Covered/glass | 32 (36.4) | | 275 (55.4) | | | 0.77 (0.63-0.95) | 0.02** |
| Door type (207 missing) | | |  | |  | | |  |  |
|  | | Wooden/metal door | 78 (88.6) | | 434 (88.4) | | | 1 |  |
|  | | Cloth/screen door | 6 (6.8) | | 38 (7.7) | | | 0.95 (0.67-1.33) | 0.77 |
|  | | No cover/other | 4 (4.6) | | 19 (3.9) | | | 1.03 (0.67-1.57) | 0.90 |
| Open eaves (199 missing) | | | 63 (71.6) | | 341 (68.3) | | | 1.02 (0.87-1.19) | 0.81 |
| Home with openings to outside (199 missing) | | | 85 (96.6) | | 477 (95.6) | | | 1.07 (0.83-1.39) | 0.59 |
| Wall construction (203 missing) | | |  | |  | | |  |  |
|  | | Cement | 28 (32.2) | | 214 (43.2) | | | 1 | - |
|  | | Corrugated metal | 25 (28.7) | | 94 (19.0) | | | 1.29 (0.96-1.74) | 0.09* |
|  | | Mud blocks | 15 (17.2) | | 130 (26.2) | | | 0.97 (0.74-1.27) | 0.84 |
|  | | Other | 19 (21.8) | | 58 (11.7) | | | 1.35 (0.96-1.88) | 0.08 |
| Floor construction (206 missing) | | |  | |  | | |  |  |
|  | | Earth/Sand | 28 (31.8) | | 167 (33.9) | | | 1 |  |
|  | | Cement | 36 (40.9) | | 251 (51.02) | | | 1.08 (0.90-1.29) | 0.40 |
|  | | Carpet tiles | 16 (18.2) | | 55 (11.2) | | | 1.27 (0.97-1.66) | 0.08* |
|  | | Other | 8 (9.1) | | 19 (3.9) | | | 1.38 (0.87-2.20) | 0.17 |
| Painted or re-plastered (199 missing) | | |  | |  | | |  |  |
|  | | >= 1 year | 39 (44.3) | | 302 (60.5) | | | 1 |  |
|  | | Never | 36 (40.9) | | 103 (20.6) | | | 1.25 (1.03-1.51) | 0.02** |
|  | | < 1 year | 10 (11.4) | | 57 (11.4) | | | 1.12 (0.84-1.51) | 0.44 |
|  | | Don’t know | 3 (3.4) | | 37 (7.4) | | | 0.90 (0.70-1.15) | 0.39 |
| Traditional home (210 missing) | | | 8 (9.20) | | 91 (18.6) | | | 0.80 (0.68-0.95) | 0.009** |
| Household level | | | | | | | | | |
| Travel to moderate endemic (5-10% PfPR2-10) | | | 24 (22.4) | | | 150 (22.1) | | 1.24 (0.76-2.03) | 0.39 |
| Travel to Angola | | | 16 (15.0) | | | 59 (8.7) | | 2.26 (1.00-5.10) | 0.05* |
| Socioeconomic PCA component (26 missing) | | | -0.17^4^ | | | 0.28^4^ | | 0.79 (0.63-0.99) | 0.04** |
|  | | squared term | - | | | - | | 1.09 (1.00-1.19) | 0.04** |
| Predicted travel time to clinic (min, 1 missing) | | |  | | |  | |  |  |
|  | | 0 - 5 | 60 (56.6) | | | 401 (59.1) | | 1 |  |
|  | | 5-15 | 43 (40.6) | | | 135 (19.9) | | 2.04 (1.10-3.78) | 0.02** |
|  | | 15 - 46 | 3 (2.8) | | | 143 (21.1) | | 0.48 (0.12-1.90) | 0.29 |
| Net coverage (1 per 2 ppl) | | | 34 (31.8) | | | 159 (23.4) | | 0.96 (0.54-1.71) | 0.90 |
| Sprayed in past year (4 missing) | | | 28 (26.4) | | | 177 (26.2) | | 1.04 (0.56-1.93) | 0.91 |
| Presence of breeding sites (3 missing) | | | 77 (*74.0*) | | | 383 (*56.4*) | | 1.77 (0.94-3.34) | 0.08* |
| Less than 15 km from Angolan border | | | 82 (76.6) | | | 354 (52.1) | | 4.50 (2.02-10.04) | <0.0001** |
| Elevation (m, 1 missing) | | |  | | |  | |  |  |
|  | | 1092-1106 | 32 (30.2) | | | 243 (35.8) | | 1 |  |
|  | | 1107-1111 | 49 (46.2) | | | 228 (33.6) | | 2.29 (1.17-4.49) | 0.02** |
|  | | 1112-1195 | 25 (23.6) | | | 208 (30.6) | | 2.39 (0.95-5.99) | 0.06* |
| Land surface temperature (C˚, 1 missing) | | |  | | |  | |  |  |
|  | | 27.5-32.2 | 32 (30.2) | | | 230 (33.9) | | 1 |  |
|  | | 32.3-39.6 | 49 (46.2) | | | 217 (32.0) | | 2.42 (1.30-4.52) | 0.006** |
|  | | 39.7-50.0 | 25 (23.6) | | | 232 (34.2) | | 1.88 (0.92-3.86) | 0.09* |
| Enhanced Vegetation Index (EVI) (1 missing) | | |  | | |  | |  |  |
|  | | 0.11-0.24 | 41 (38.3) | | | 486 (71.6) | | 1 |  |
|  | | 0.25-0.34 | 58 (54.2) | | | 147 (21.7) | | 5.39 (2.58-11.27) | <0.0001** |
|  | | 0.35-0.45 | 8 (7.5) | | | 46 (6.7) | | 1.90 (0.51-7.02) | 0.33 |
| Total rainfall in prior month (mm; 1 missing) | | |  | | |  | |  |  |
|  | | 0 | 16 (15.1) | | | 217 (31.8) | | 1 |  |
|  | | 0.1-19.9 | 67 (63.2) | | | 183 (26.8) | | 6.61 (3.17-13.81) | <0.0001 |
|  | | 20 – 67 | 23 (21.7) | | | 283 (41.4) | | 2.04 (0.88-4.76) | 0.10 |
| Distance to waterbody (km; 1 missing) | | |  | | |  | |  |  |
|  | | 0 – 4.9 | 90 (84.9) | | | 462 (68.0) | | 1 |  |
|  | | 5.0 – 9.9 | 13 (12.3) | | | 103 (15.2) | | 0.88 (0.39-2.00) | 0.77 |
|  | | 10.0 – 13.1 | 3 (2.8) | | | 114 (16.8) | | 0.98 (0.21-4.56) | 0.98 |
| Community level | | | | | | | | | |
| High transmission season | | | 84 (78.5) | 405 (59.7) | | | | 4.58 (2.46-8.53) | <0.0001** |
| GEE: Generalized estimating equations; CI: confidence interval; QIC: quasilikelihood under the independence model criterion; m: meters; C˚: degrees Celsius; mm: millimeters; km: kilometers; PfPR2-10: mean annually averaged prevalence of P falciparum infection in 2-10 year olds  ** P-value ≤ 0.05; * P-value ≤ 0.10  ^1^ One hundred and seven cases and 679 controls are included, minus the number of missing data points shown for each variable.  ^2^ P-values from wald tests.  ^3^ Excluded from analysis due to perfect prediction of failure in cases.  ^4^ Mean value of continuous variable | | | | | | | | | |
