# Supplementary material for: Malaria risk in young male travellers but local transmission persists: a case–control study in low transmission Namibia
Source: Malar J. 2017 Feb 10;16:70. doi: 10.1186/s12936-017-1719-x (PMC5303241; doi:10.1186/s12936-017-1719-x)
Supplement: Supplementary file 4 — Additional file 4. Sensitivity analysis for modification of the effect of travel to Angola on malaria by gender in adjusted analyses1. [file 12936_2017_1719_MOESM4_ESM.docx]

| Table S4. Sensitivity analysis for modification of the effect of travel to Angola on malaria by gender in adjusted analyses^1^ | | | | | |
| --- | --- | --- | --- | --- | --- |
|  | No Travel to Angola | | Travel to Angola | | Adjusted ORs (95% CI) for travel within gender strata |
|  | N with/without malaria | Adjusted OR (95% CI) | N with/without malaria | Adjusted OR  (95% CI) |  |
| Sensitivity Analysis 1 | | | | | |
| Female | 34/272 | 1.0 | 3/8 | 1.06 (0.89-1.28) | 1.06 (0.89-1.28) |
| Male | 46/184 | 1.05 (1.00-1.09) | 15/2 | 2.78 (0.49-15.83) | 2.65 (0.47-15.00) |
| Measure of effect modification on additive scale: RERI (95% CI) = 1.66 (-3.2-6.5); P=0.50^3^  Measure of effect modification on multiplicative scale: ratio of ORs (95% CI) = 2.60 (0.45-14.9); P=0.28 | | | | | |
| Sensitivity Analysis 2 | | | | | |
| Female | 34/328 | 1.0 | 3/13 | 1.56 (0.36-6.79) | 1.56 (0.36-6.79) |
| Male | 46/247 | 1.91 (1.22-2.97) | 15/3 | 77.82 (4.13-1467) | 40.83 (2.15-775) |
| Measure of effect modification on additive scale: RERI (95% CI) = 75.35 (-153-303); P=0.52^3^  Measure of effect modification on multiplicative scale: ratio of ORs (95% CI) = 51.60 (1.84-1444); P=0.02 | | | | | |
| ^1^ GEE adjusted for health district (matching variable), clustering of controls within households and covariates listed in Additional file 5 (Table S5)  ^3^ Confidence intervals calculated using Delta approximation | | | | | |
