# Supplementary material for: Malaria risk in young male travellers but local transmission persists: a case–control study in low transmission Namibia
Source: Malar J. 2017 Feb 10;16:70. doi: 10.1186/s12936-017-1719-x (PMC5303241; doi:10.1186/s12936-017-1719-x)
Supplement: Supplementary file 5 — Additional file 5. Sensitivity analysis for multivariate logistic regression model1. [file 12936_2017_1719_MOESM5_ESM.docx]

| Table S5 Sensitivity analysis for multivariate logistic regression model^1^. | | | | | | | | | |
| --- | --- | --- | --- | --- | --- | --- | --- | --- | --- |
|  | | Sensitivity Analysis 1 | | | | Sensitivity Analysis 2 | | | |
|  | | Number^2^ | |  |  | Number^2^ | |  |  |
|  | | Case  N=98 | Control  N=466 | Adjusted OR  (95% CI) | P value | Case  N=98 | Control  N=591 | Adjusted OR  (95% CI) | P value |
| **Variable** | |  |  |  |  |  |  |  |  |
| Age group (years) | |  |  |  |  |  |  |  |  |
|  | <5 | 9 | 84 | 1 | - | 9 | 97 | 1 | - |
|  | 5-14 | 23 | 105 | 1.08 (1.03-1.13) | 0.03 | 23 | 202 | 1.48 (0.66-3.33) | 0.34 |
|  | 15-29 | 37 | 124 | 1.08 (1.04-1.13) | <0.0001 | 37 | 138 | 2.15 (0.99-4.63) | 0.05 |
|  | 30 -44 | 14 | 61 | 1.02 (0.94-1.10) | 0.65 | 14 | 68 | 2.21 (0.87-5.62) | 0.10 |
|  | 45-59 | 8 | 35 | 1.11 (0.99-1.24) | 0.07 | 8 | 28 | 3.61 (1.27-10.24) | 0.02 |
|  | 60+ | 7 | 57 | 0.99 (0.94-1.04) | 0.62 | 7 | 58 | 1.23 (0.55-2.76) | 0.61 |
| Location slept previous night | |  |  |  |  |  |  |  |  |
|  | In household | 87 | 458 | 1 | - | 87 | 583 | 1 | - |
|  | Away from household | 11 | 8 | 1.36 (1.02-1.82) | 0.04 | 11 | 8 | 6.12 (1.99-18.88) | 0.002 |
| Higher socioeconomic status^3^ | | 98 | 466 | 0.65 (0.48-0.88) | 0.005 | 98 | 591 | 0.66 (0.48-5.53) | 0.02 |
|  | squared term | - | - | 1.15 (1.04-1.26) | 0.006 | - | - | 1.12 (1.00-1.26) | 0.06 |
| Predicted travel time to clinic | |  |  |  |  |  |  |  |  |
|  | 0 - 4 | 52 | 358 | 1 | - | 52 | 370 | 1 | - |
|  | 5-14 | 43 | 92 | 1.27 (0.56-2.89) | 0.56 | 43 | 104 | 2.26 (0.92-5.53) | 0.07 |
|  | 15 - 46 | 3 | 16 | 1.62 (0.30-8.78) | 0.57 | 3 | 117 | 0.06 (0.005-0.64) | 0.02 |
| More than 15km from Angolan border | | 23 | 206 | 1 | - | 23 | 286 | 1 | - |
| Less than 15 km from Angolan border | | 75 | 260 | 3.30 (1.31-8.32) | 0.01 | 75 | 305 | 2.89 (1.19-7.02) | 0.02 |
| Enhanced Vegetation Index (EVI) ^3^ | |  |  |  |  |  |  |  |  |
|  | 0.11-0.24 | 37 | 343 | 1 | - | 37 | 400 | 1 | - |
|  | 0.25-0.34 | 54 | 90 | 21.67 (5.12-91.72) | <0.0001 | 54 | 145 | 12.37 (3.23-47.30) | <0.0001 |
|  | 0.35-0.45 | 7 | 33 | 3.66 (0.82-16.43) | 0.09 | 7 | 46 | 1.68 (0.19-14.76) | 0.64 |
| Total rainfall in prior month (mm) | |  |  |  |  |  |  |  |  |
|  | 0 – 19 | 14 | 196 | 1 | - | 14 | 175 | 1 | - |
|  | 20 - 39 | 63 | 154 | 3.59 (1.47-8.78) | 0.005 | 63 | 160 | 2.60 (0.94-7.24) | 0.07 |
|  | 40 - 67 | 21 | 116 | 0.36 (0.08-1.66) | 0.18 | 21 | 256 | 0.24 (0.06-0.91) | 0.04 |
| District: Engela | | 51 | 253 | 1 | - | 51 | 252 | 1 | - |
|  | Oshikuku^4^ | 21 | 66 | 1.49 (0.49-4.58) | 0.48 | 21 | 70 | 4.91 (1.35-17.77) | 0.02 |
|  | Outapi | 26 | 147 | 0.46 (0.20-1.04) | 0.06 | 26 | 269 | 1.13 (0.44-2.89) | 0.80 |
| OR: odds ratio; GEE: Generalized estimating equations; CI: confidence interval; QIC: quasilikelihood under the independence model criterion; m: meters; C˚: degrees Celsius; mm: millimeters; km: kilometers  ^1 A^djusted for health district (matching variable), clustering of controls within households and interaction effect of gender and travel shown in Additional file 5 (Table S4)  ^2^ Numbers restricted to non-missing data in final multivariate model in (Table 3 & 4)  ^3^ Socioeconomic measure is first component of the PCA, included as a continuous measure  ^4^ The higher adjusted odds of malaria observed in Oshikuku is attributed to a lower number of controls recruited per case in this area compared to the other districts. | | | | | | | | | |
